# CAMDI interacts with the human memory-associated protein KIBRA and regulates AMPAR cell surface expression and cognition

**DOI:** 10.1371/journal.pone.0224967

**Published:** 2019-11-15

**Authors:** Toshifumi Fukuda, Shun Nagashima, Ryoko Inatome, Shigeru Yanagi

**Affiliations:** Laboratory of Molecular Biochemistry, School of Life Sciences, Tokyo University of Pharmacy and Life Sciences, Hachioji, Tokyo, Japan; State University of New York Downstate Medical Center, UNITED STATES

## Abstract

Little is known about the molecular mechanisms of cognitive deficits in psychiatric disorders. CAMDI is a psychiatric disorder-related factor, the deficiency of which in mice results in delayed neuronal migration and psychiatrically abnormal behaviors. Here, we found that CAMDI-deficient mice exhibited impaired recognition memory and spatial reference memory. Knockdown of CAMDI in hippocampal neurons increased the amount of internalized alpha-amino-3-hydroxy-5-methyl-4-isoxazole propionate receptor (AMPAR) and attenuated the chemical long-term potentiation (LTP)-dependent cell surface expression of AMPAR. KIBRA was identified as a novel CAMDI-binding protein that retains AMPAR in the cytosol after internalization. KIBRA inhibited CAMDI-dependent Rab11 activation, thereby attenuating AMPAR cell surface expression. These results suggest that CAMDI regulates AMPAR cell surface expression during LTP. CAMDI dysfunction may partly explain the mechanism underlying cognitive deficits in psychiatric diseases.

## Introduction

Modification of synaptic strength thought to contribute to learning and memory is called synaptic plasticity. The most widely studied form of synaptic plasticity is long-term potentiation (LTP). An understanding of the cellular and molecular mechanisms of alpha-amino-3-hydroxy-5-methyl-4-isoxazole-propionate receptor (AMPAR) trafficking would increase our understanding of LTP. LTP induction leads to an increase in the number of functional AMPARs at post-synaptic cell surfaces [1, 2]. Synaptic strength is determined, in part, by the expression level of AMPARs at synapses [3]. AMPARs are mobilized to the recycling endosomal compartment by synaptic activity, and they are further exocytosed from recycling endosomes (REs) to the postsynaptic membrane by LTP induction [4, 5]. Rapid translocation of REs to dendritic spines is required for synaptic strength through an increase in the number of surface AMPARs [6]. Several regulators for recycling endocytosis of AMPAR have been identified so far [6–9]. Among these, kidney and brain expressed protein (KIBRA) has been shown to control endocytic recycling of transferrin receptor (TfR) and AMPAR [10]. Indeed, KIBRA knockout (KO) mice have severe deficits in contextual fear learning and memory, indicating that KIBRA is a pivotal regulator of AMPAR trafficking during LTP or LTD [11, 12]. However, the molecular mechanism underlying AMPAR trafficking regulation by KIBRA remains largely unknown.

We previously identified a DISC1-interacting protein, named CAMDI (Coiled-coil protein Associated with Myosin II and DISC1), which regulates cortical neuronal migration in brain development [13, 14]. CAMDI KO mice show delayed cortical migration and abnormal behaviors associated with psychiatric disorders, including hyperactivity, repetitive behaviors, and grooming and social abnormalities observed in autism patients [15]. Furthermore, analyses of the results of a recent genome-wide association study (GWAS) suggest that the SNP in the CAMDI gene is linked to some extent to psychiatric diseases [16, 17], although the change in CAMDI expression from the polymorphisms that give the GWAS effect are not known..

Cognitive deficits are often observed in psychiatric disorders [18], but the mechanisms accounting for these relationships are not clear. In this study, by analyzing CAMDI KO mice, we found a critical role of CAMDI in learning and memory performance through regulation of AMPAR cell surface expression in competition with KIBRA.

## Materials and methods

### Mice

CAMDI KO mice were produced in previously described [15]. Male mice between the ages of 8–10 weeks were used for behavior studies. The experimenters were blind of the genotype of the tested animals for data collection and analyses. The number of mice in each behavioral test was different because different groups of mice were used for the behavioral tests.

All animals were maintained under the university guidelines for the care and use of animals. The experiments were performed after securing Tokyo University of Pharmacy and Life Sciences Animal Use Committee Protocol approval.

### Antibodies

Anti-CAMDI antibody was produced by immunizing a rabbit with synthetic peptide in previously described [13]. Anti-KIBRA antibody was produced by immunizing a rabbit with synthetic peptide (RRRLEKDLQAARDTQSK). Anti-FLAG M2 monoclonal and anti-α-tubulin antibody were obtained from Sigma-Aldrich. Anti-HA high affinity antibody obtained from Roche. Anti-GFP rabbit polyclonal antibody and secondary antibodies conjugated with Alexa Fluor 350, 488, 594 were obtained from Invitrogen. Anti-GFP mouse monoclonal and Anti-DsRed polyclonal antibody were purchased from Clontech. Anti-surface GluA2 antibody was purchased from Chemicon.

### Plasmids

The mouse full-length CAMDI was described previously [13]. A plasmid for CAMDI-sh and sh-resistant construct were previously described [13]. The mouse KIBRA was derived from RT-PCR product of mouse brain total RNA. The forward primer (5’- GCGCGAATTCATGCCCCGGCCGGAGTTGCC-3’), the reverse primer (5’- GGGGCTCGAGTTAGACGTCATCTGCAGAG-3’). KIBRA-sh3 sequence is shown below (with an order of sense, loop (underlined), and antisense): 5’- GCACAAGAGTGAGTTGCAAGCTTCAAGAGAGCTTGCAACTCACTCTTGTGC-3’. Constructs of HA-tagged CAMDI coiled-coil domains (CC1, 576–706; CC2, 707–906; CC3, 907–1,106 amino acids) were generated by PCR using previously described FLAG-CAMDI constructs [13]. The dominant negative Rab11 construct (S25N) and dominant active Rab11 construct (Q70L) was generated from the wild-type EGFP-Rab11 construct. The rat Rab11a was derived from RT-PCR product of rat brain total RNA.

### Cell culture and transfections

SH-SY5Y cells were obtained from ATCC. These cells were maintained in Dulbecco’s minimal essential medium supplemented with 10% fetal bovine serum at 37 °C, in 5% CO_2_, in a humidified chamber. Transfection was carried out using Lipofectamine 2000 (Invitrogen). Primary hippocampal neurons were prepared from ICR mice (embryonic day 18) and plated on a poly-L-Lysine coated slide glass in Minimum Essential Medium Eagle containing 2% FBS and N2 supplement. Transfection was carried out using Lipofectamine LTX and Lipofectamine 2000 (Invitrogen).

### Immunoprecipitation and western blotting

Cultured cells were lysed in NP-40 lysis buffer (20 mM Tris-HCl, pH7.2, 2 mM EDTA, 0.5% NP-40, 8% sucrose, 80 mM dithiothreitol). The lysate was clarified by centrifugation at 15,000 *g* for 10 min at 4 °C. The supernatant was incubated with antibody and mixed with protein A- or G-sepharose beads (GE Healthcare). Immunoprecipitates were washed three times with lysis buffer. After boiling for 3 min, equal protein amounts of the lysates were subjected to SDS-PAGE and transferred to polyvinylidene difluoride membranes (Immobilon P, Millipore). Membrane were blocked for 1 hour at room temperature in 5% skim milk in PBST with gentle shaking and incubated with primary antibodies overnight at 4 °C. After washing the membrane three times with PBST, they were incubated with secondary antibody conjugated to horseradish peroxidase for 1 h at room temperature. The blotted membrane was visualized using the Immobilon Western chemiluminescent HRP substrate (Millipore) according to the manufacturer’s instructions.

### Immunofluorescence

Cells were fixed for 20 min in PBS containing 4% paraformaldehyde and permeabilized with 0.2% Triton X-100. After incubation in PBS containing 1% bovine serum albumin for 30 min, the cells were reacted with 1st antibody for overnight at 4 °C, followed by incubation with secondary antibody. The staining was analyzed by confocal microscope (OLYMPUS FV1000-D).

### Yeast two-hybrid screening

The Matchmaker two-hybrid system kit (Clontech) was used for detecting specific proteins interacting with CAMDI coiled-coil region as described by manufacturer.

### AMPAR internalization assay

Neurons were transfected with indicated plasmids and EGFP plasmid for 72 h. Surface GluA2 was labeled with mouse anti-surface GluA2 antibody (5 μg/ml) for 10 min at 37 °C. Neurons were incubated for 1 h at 37 °C to allow internalization of AMPAR. After fixation, labeled-GluA2 reinserted into the surface was blocked under nonpermeabilizing conditions with unlabeled mouse secondary antibody. Neurons were then permeabilized and incubated with Alexa 594-conjugated secondary antibody to label the remaining intracellular internalized GluA2. Fluorescence signal was quantified with FLA-9000 (Fuji film).

### Glycine-induced AMPAR insertion assay

Insertion assay was performed as described previously [4]. In brief, neurons were transfected with indicated plasmids and EGFP for 72 h. Surface GluA2 subunit was labeled with mouse anti-surface GluA2 antibody (5 μg/ml) for 10 min at 37 °C and stimulated with or without glycine (200 μM, 3 min, room temperature) in extracellular solution containing (mM): 150 NaCl, 2 CaCl2, 5 KCl, 10 HEPES, 30 glucose, 0.0005 TTX, 0.001 strychnine, 0.02 bicuculline methiodide (pH 7.4). Neurons were then incubated in the same extracellular solution without any added glycine for 25 min at 37 °C to allow for recycling of receptors. Neurons were fixed, washed, and labeled with Alexa 594-conjugated secondary antibody to visualize surface GluA2 subunit.

### Image analysis and quantification

For GluA2 image quantification, regions of interest dendrites were selected blind based on GFP fluorescence. The total intensity of GluA2 fluorescence were determined using ImageJ in a square of about 3×25 μm region of secondary dendrite and the background intensity subtracted from the intensity of the regions. GluA2 fluorescence intensity was normalized by the co-transfected EGFP fluorescence intensity. Pearson’s correlation coefficients were measured by using ImageJ/Fiji software. Images were acquired with FV1000-D (OLYMPUS).

### Novel object recognition test

The testing apparatus was a white, plastic transport box (40×40×15 cm). After 5 min of habituation to the box, mice explored two objects for memory consolidation for 10 min, after which they were returned to their home cage. 5 min or 24 h after initial object exposure, one object was replaced by novel object and the mice were returned to testing chamber for assessment of object recognition memory for another 10 min. The duration of exploration of each object as well as total object exploration time was recorded. Data are presented as a recognition index (time attending to object B/time attending to object A + B).

### Social recognition test

The testing apparatus was a white, plastic transport box (40×40×15 cm). Test mouse was placed in the box and allowed habituate for 10 min, after which a visitor mouse was introduced into the box. After 5 min, visitor mouse was removed. After a 10 min inter-exposure interval, the same stimulus mouse was reintroduced. We repeated this sequence for three trials. In a fourth ‘dishabituation’ trial, the test mice were then exposed to a novel stimulus mouse for 5 min. The time the test mouse spent interacting with each visitor mouse was recorded. Social recognition was measured by social interaction time.

### Barnes maze test

Barnes maze test was performed according to Amador-Arjona et al. [19]. The Barnes maze consists of a circular platform 90 cm in diameter elevated 43 cm above the floor by a tripod. Twelve holes (diameter 11.5 cm) were equidistantly located around the perimeter and centered 5 cm from it. A dark escape box [19×13×6 cm] was placed under only one hole randomly chosen for each mouse. Mice were placed under the chamber in the center of the maze for 1 min then given 3 min to locate the escape box freely. If the mice did not find the correct hole, they were gently directed toward the target hole and allowed to descend into the escape box 1 min. The time to reach the target hole and number of errors were recorded. Total errors (put the face in the other hole during 180 s of test) before the animal enters the target hole were quantified. Mice were tested once a day for 12 d for the acquisition portion of the study (training session). On the day after training session, the escape box was removed and the mouse was allowed to explore the maze for 3 min (probe test). Mice were then retested 2 weeks after probe test with the escape box at the original position in order to examine retention of spatial memory. On the day after this test, the escape box was moved 180° around the maze, and the mouse was allowed to freely explore the maze for 3 min (reversal test). Sessions were videotaped and scored by an experienced observer blind to mouse genotype. Data are presented as the percent time of visits to the target hole and those directly adjacent (1st left and right, 90°) or 270° apart.

### Statistical analysis

Two-way ANOVA with repeated measures followed up by Scheffe’s post-hoc test or Tukey HSD test were used to examine group differences. Data are presented as mean ±SEM. Student’s t-test was applied to test the significance of differences between mean values where factorial ANOVA was not required.

## Results

### CAMDI KO mice show reduced recognition memory

**Fig 1:**
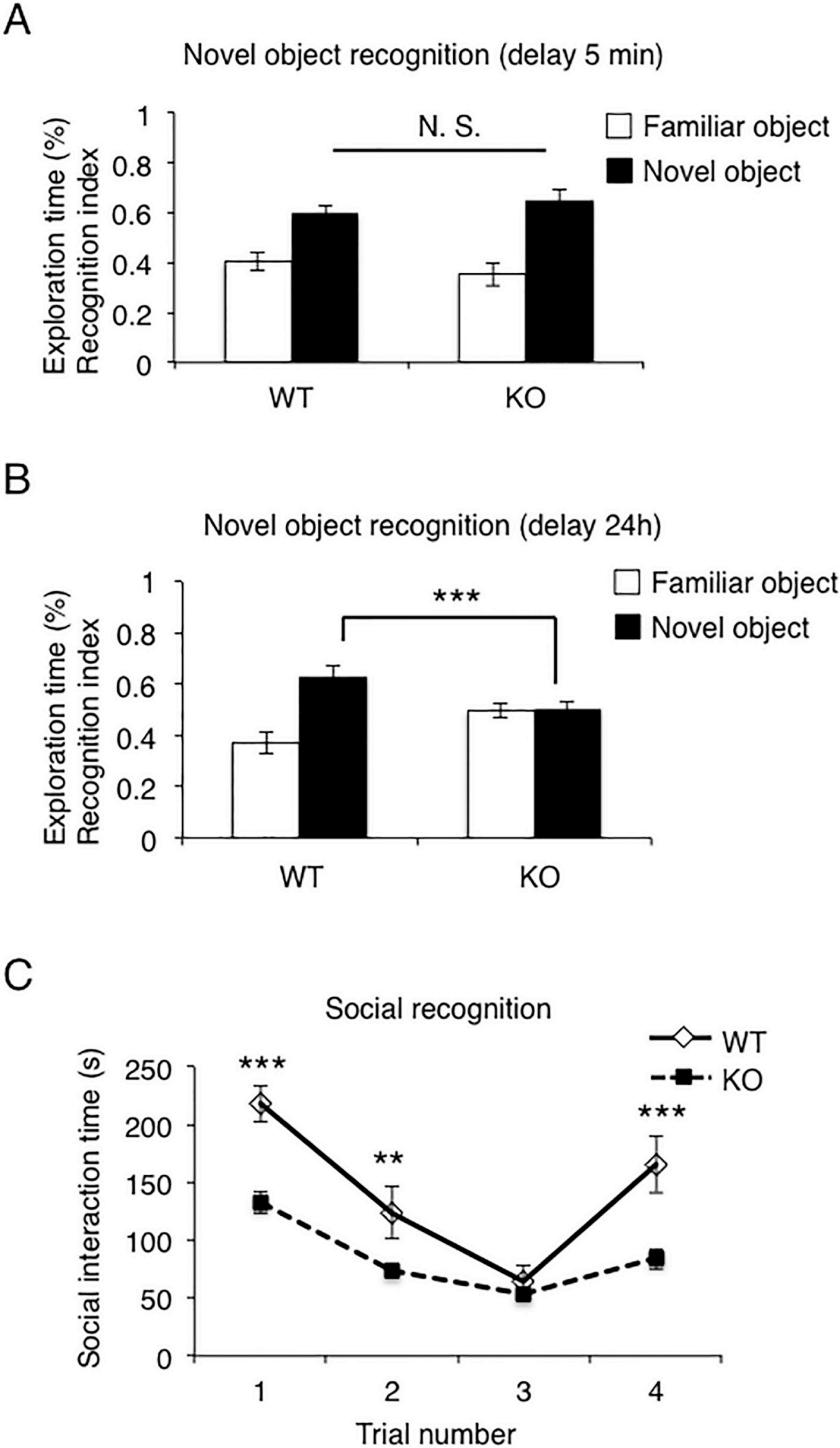
Impaired novel object and social recognition memory in CAMDI KO mice. (A) Short-term memory (delay 5 min) was comparable between WT and CAMDI KO mice. n = 6 for WT mice, n = 8 for KO mice. N. S., not significant. Two-way ANOVA followed by Scheffe’s post-hoc test. Data are presented as mean ± SEM. (B) Long-term memory (delay 24 h) was impaired in CAMDI KO mice. n = 9 for WT mice, n = 9 for KO mice. ***, p<0.001, Two-way ANOVA followed by Scheffe’s post-hoc test. Data are presented as mean ± SEM. (C) Social recognition memory was impaired in CAMDI KO mice. Trials 1 to 3 represent repeated presentation of the same stimulus mouse; trial 4 represents introduction of a novel stimulus mouse. n = 9 for WT mice, n = 9 for KO mice. **, p<0.01, ***, p<0.001, Two-way ANOVA followed by Scheffe’s post-hoc test. Data are presented as mean ± SEM.

Yeast two-hybrid screening using CAMDI as bait identified KIBRA, which has been shown to regulate learning and memory. This promoted us to investigate whether CAMDI KO mice would suffer from memory disturbance. We first performed a novel object recognition test—a non-spatial memory task that relies on the natural exploratory behavior of mice. Mice were exposed to objects for 10 min, and after 5 min or 24 h interval, the mice were re-exposed to objects, and then one of the familiar objects was replaced with a novel object. The times spent exploring the different objects were measured. During habituation, there was no difference in exploration time between WT and CAMDI KO mice, suggesting that they were equally motivated to explore objects (S1A Fig). When one of the familiar objects was replaced with a novel object after 5 min, no significant difference in time exploring the novel object was observed between the genotypes (Fig 1A). However, when the novel object was introduced after 24 h, WT mice spent significantly more time than CAMDI KO mice exploring the new object (Fig 1B). These results indicated that CAMDI is necessary for the formation of the memory.

To understand impaired recognition memory from a different perspective, mice were subjected to a social-recognition test (Fig 1C). WT mice showed a significant decrease in the time spent sniffing a similar mouse, suggesting normal social recognition and intact social short-term memory. In contrast, during the first 5 min after presentation of the new mouse, CAMDI KO mice showed reduced social interaction time, possibly because of poor sociability, as previously reported [15]. Notably, in CAMDI KO mice, no significant difference in social interaction time was observed between the second and third trials, which involved reintroduction of the same visitor mouse as used in the first trial, for 5 min each time, with 10 min inter-exposure intervals. When, in the fourth trial, a different visitor mouse was introduced, a significant increase in social interaction was observed in WT mice. However, CAMDI KO mice showed a low level of social interaction with this second new visitor within groups (Fig 1C). Next, we compared the ratio of trial 3 to trial 1 as an indication of whether the repeated presentation of the first mouse is recognized as familiar with repeated exposure. As a result, we showed the ratio of trial 3 to trial 1 increased significantly in CAMDI KO mice (S1B Fig). Together, these results indicate an impairment of social recognition memory in CAMDI KO mice.

### CAMDI KO mice show impairment of spatial reference memory

**Fig 2:**
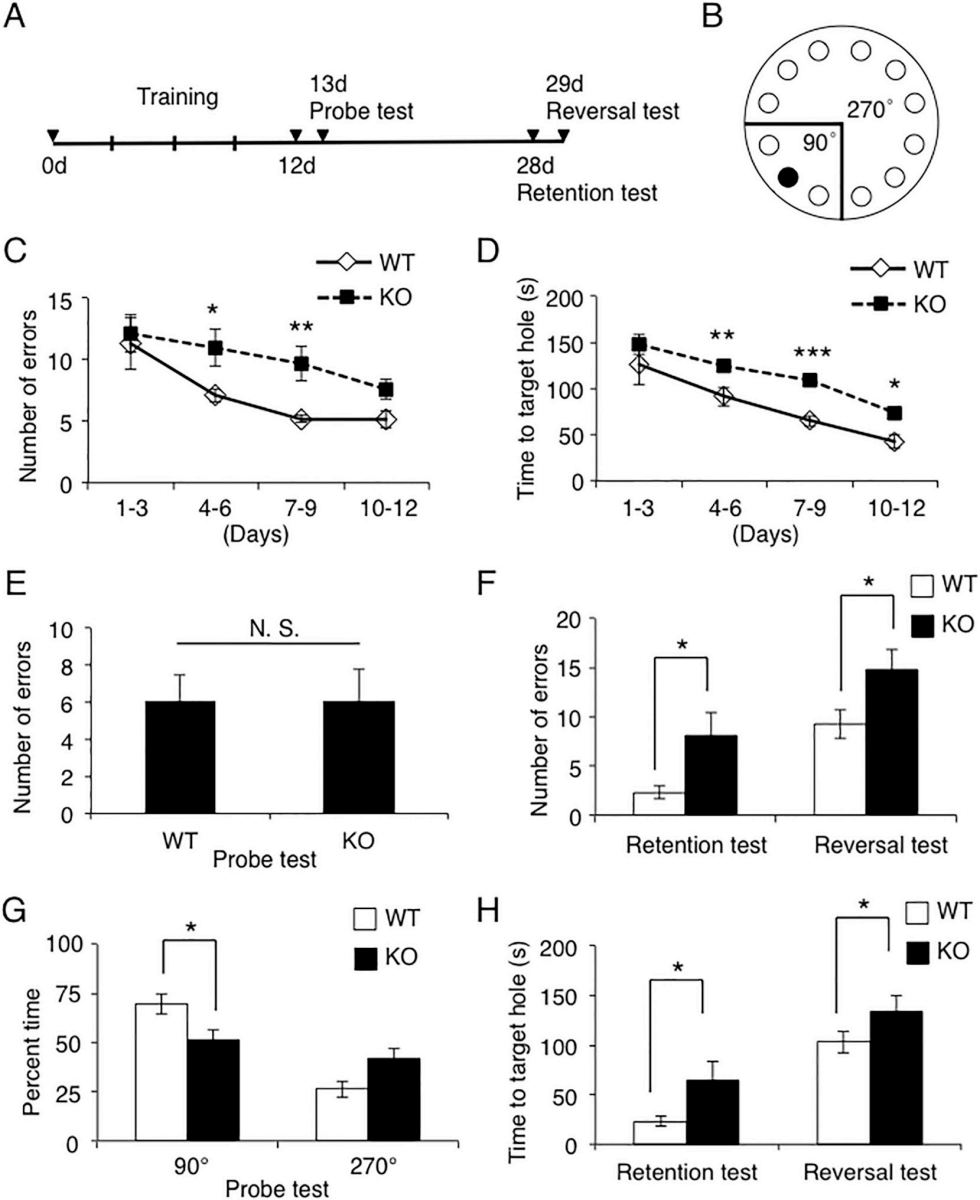
Impaired spatial learning and memory formation for Barnes test in CAMDI KO mice. (A) Experimental design of Barnes maze test. (B) Barnes maze diagram. (C) Error rates in training session (Day1–12). n = 13 for WT mice, n = 14 for KO mice. *, p<0.05, **, p<0.01 (WT vs. KO mice), Two-way ANOVA followed by Scheffe’s post-hoc test. Data are presented as mean ± SEM. (D) CAMDI KO mice had longer times to target hole in training session. n = 13 for WT mice, n = 14 for KO mice. *, p<0.05, **, p<0.01, ***, p<0.001 (WT vs. KO mice), Two-way ANOVA followed by Scheffe’s post-hoc test. Data are presented as mean ± SEM. (E) Number of errors on the hole before removing the escape box in the probe test at one day after the last training day. n = 13 for WT mice, n = 14 for KO mice. N. S., not significant, Student’s t-test. Data are presented as mean ± SEM. (F) Number of errors in the retention test at 2 weeks after the last training day and reversal test at one day after retention test. n = 13 for WT mice, n = 14 for KO mice. *, p<0.05, Student’s t-test. Data are presented as mean ± SEM. (G) Percent time in the probe test at one day after the last training day. n = 13 for WT mice, n = 14 for KO mice. *, p<0.05, Two-way ANOVA followed by Scheffe’s post-hoc test. Data are presented as mean ± SEM. (H) Time to target hole in the retention test at 2 weeks after the last training day and reversal test at one day after retention test. n = 13 for WT mice, n = 14 for KO mice. *, p<0.05, Student’s t-test. Data are presented as mean ± SEM.

We next performed the Barnes maze test, a hippocampus-dependent cognitive task that requires spatial reference memory. Mice were subjected to 12 trials of spatial training that required them to learn the location of a hidden escape box under a 12-hole platform. The number of errors and the time taken to find the target hole were assessed in accordance with the steps and methods shown in Fig 2A and 2B. Both WT and CAMDI KO mice showed a decreasing trend in the number of errors and the time taken to reach the target hole during training, suggesting that both mice have learned to use the spatial cues to find the hidden target (Fig 2C and 2D). However, both the number of errors and the time taken to reach the target hole at 4 to 6 and 7 to 9 days were significantly greater in CAMDI KO mice than in WT mice. These data suggest that there is a defective in spatial learning performance in CAMDI KO mice.

Spatial memory formation was assessed by using a probe test in which the escape box was removed on day 13. Compared to WT mice, CAMDI KO mice are remembering the same amount of information of the spatial cues to find the hidden target on day 13 (Fig 2E). On the other hand, CAMDI KO mice spent significantly less time than WT mice in the target quadrant including the target and adjacent holes within 90° (Fig 2G). Conversely, CAMDI KO mice tended to spend more time near the opposite holes (i.e. around 270°), although the between group difference was not statistically significant. Thus, CAMDI KO mice showed a mild reduction in spatial memory formation.

Long-term spatial memory was then assessed 2 weeks after the last trial. CAMDI KO mice showed a significant increase in the number of errors on the retention test relative to the control group (Fig 2F, left). A reversal test, in which the hidden platform was moved to a new location, was performed after the retention test. CAMDI KO mice showed a significant increase in the number of errors on the reversal test relative to the control group (Fig 2F, right). CAMDI KO mice also showed a significant increase in the time to target hole on the retention test and reversal test (Fig 2H). Thus, CAMDI KO mice showed a significant reduction in long-term spatial memory. These results demonstrated that CAMDI was required for spatial learning and reference memory formation and consolidation.

### CAMDI regulates the amount of internalized AMPAR

**Fig 3:**
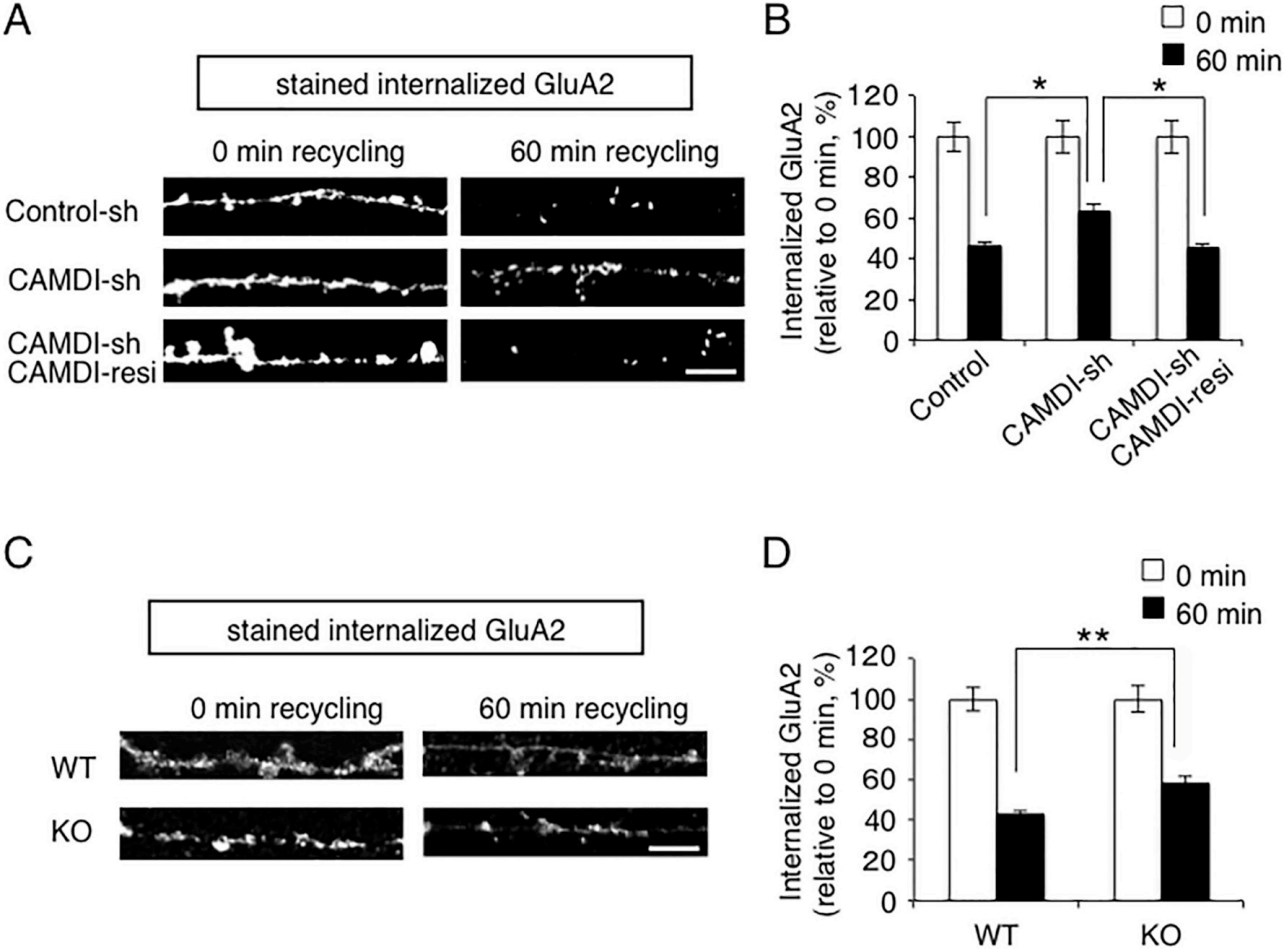
CAMDI regulates the amount of internalized AMPAR. (A) Knockdown of CAMDI increased the amount of internalized AMPAR. Antibody-feeding assay to monitor AMPAR expression (see Materials and methods) was performed on hippocampal neurons transfected at DIV17 with indicated plasmid(s) and EGFP plasmid. Fluorescence signal indicates remaining intracellular GluA2 subunit after 0 min and 60 min at DIV21. Scale bar indicates 5 μm. (B) Internalized intracellular GluA2 subunit on individual neurons from (A). The average GluA2 fluorescence intensity in the dendritic segment was normalized to the dendritic length (10 μm) for each experimental condition. Control, 0 min, n = 14; Control, 60 min, n = 11; CAMDI-sh, 0 min, n = 15; CAMDI-sh, 60 min, n = 15; CAMDI-sh and CAMDI-resistance, 0 min, n = 11; CAMDI-sh and CAMDI-resistance, 60 min, n = 12. *, p<0.05, Two-way ANOVA followed by Tukey HSD test. Data are presented as mean ± SEM. (C) CAMDI KO mice regulated amount of internalized AMPAR. Antibody-feeding assay to monitor AMPAR internalization was performed on hippocampal neurons from WT and CAMDI KO mice. Fluorescence signal indicates remaining intracellular GluA2 subunit after 0 min and 60 min. Scale bar indicates 5 μm. (D) Internalized intracellular GluA2 subunit on individual neurons from (C). The average GluA2 fluorescence intensity in the dendritic segment was normalized to the dendritic length (10 μm) for each experimental condition. Control, 0 min, n = 29; Control, 60 min, n = 28; CAMDI KO, 0 min, n = 32; CAMDI KO, 60 min, n = 28. **, p<0.01, Two-way ANOVA followed by Tukey HSD test. Data are presented as mean ± SEM.

AMPAR movement plays a key role in the molecular mechanisms of learning and memory. Since CAMDI is highly expressed in the hippocampus [13] and interacts with KIBRA, a regulator of AMPAR recycling, we next investigated whether CAMDI is involved in AMPAR regulation in cultured hippocampal neurons. For this purpose, surface GluA2 was labeled with mouse anti-surface GluA2 antibody for 10 min and then incubated for 60 min to allow internalization of cell surface receptors [20]. After fixation of neurons, labeled-GluA2 reinserted into the surface was blocked under non-permeabilizing conditions with unlabeled mouse secondary antibody. Neurons were then permeabilized and incubated with Alexa 594-conjugated secondary antibody to label the remaining intracellular internalized GluA2. Quantitative analysis of fluorescence signals indicated that internalized GluA2 subunit was retained in the intracellular compartment in CAMDI-sh transfected neurons, whereas a major part of GluA2 subunit was localized on the cell surface within 60 min in control-sh-transfected neurons (Fig 3A and 3B), suggesting that the amount of internalized GluA2 subunit was increased by CAMDI knockdown.

To demonstrate the specific effect of CAMDI-sh in GluA2 regulation, a rescue experiment was performed by expression of a CAMDI-sh-resistant CAMDI mutant [13]. As expected, transfection with CAMDI-sh and CAMDI-sh-resistant gene (CAMDI-resi) completely rescued the perturbation of the amount of internalized GluA2 subunit (Fig 3A and 3B).

To further confirm the essential role of CAMDI in GluA2 regulation, we compared the amount of internalized GluA2 that remained intracellular between primary cultured hippocampal neurons derived from WT mice and from CAMDI KO mice. A significant increase in internalized GluA2 subunit was observed in CAMDI-deficient neurons compared with WT neurons at 60 min (Fig 3C and 3D), suggesting that AMPAR expression on the cell surface was reduced under basal conditions. However, there was no significant difference in surface AMPAR expression at 0 min in CAMDI KO mice (S2 Fig, discussed in below). Taken together, these results indicated that CAMDI regulated the amount of internalized GluA2 subunit in hippocampal neurons.

### CAMDI regulates LTP-dependent cell surface expression of AMPAR

**Fig 4:**
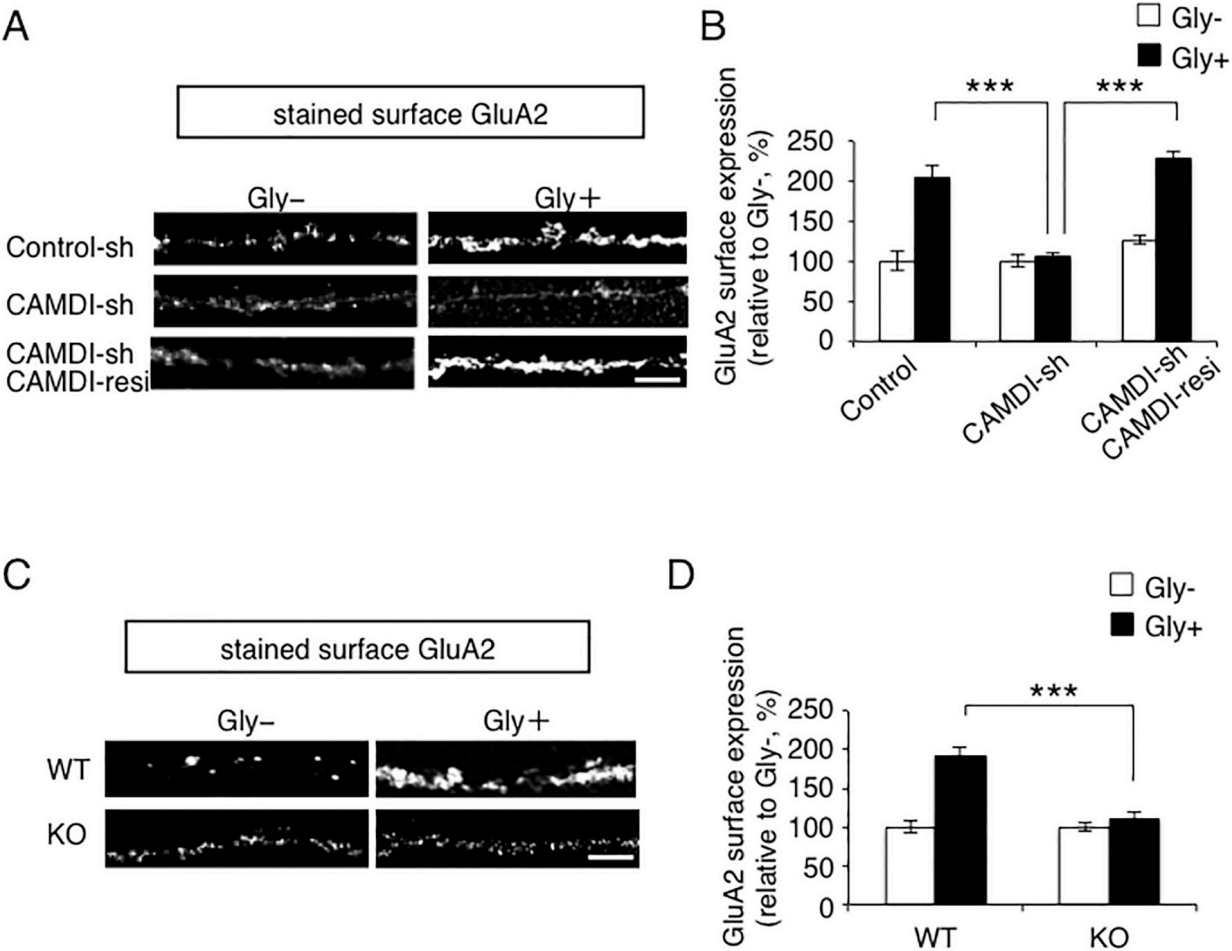
CAMDI regulates cLTP-induced cell surface expression of AMPAR. (A) Knockdown of CAMDI inhibited cLTP-induced cell surface expression of AMPAR. To monitor AMPAR cell surface expression, antibody-feeding assay was performed on hippocampal neurons transfected with indicated plasmid and EGFP plasmid at DIV17. Neurons were stimulated with or without glycine and incubated at DIV21. Scale bar, 5 μm. (B) GluA2 cell surface expression on individual neurons from (A). The average GluA2 fluorescence intensity was normalized to the dendritic length (10 μm) for each experimental condition. Control, Gly-, n = 22; Control, Gly+, n = 10; CAMDI-sh, Gly-, n = 18; CAMDI-sh, Gly+, n = 11; CAMDI-sh, CAMDI-resistance, Gly-, n = 10; CAMDI-sh, CAMDI-resistance, Gly+, n = 10. ***, p<0.001, Two-way ANOVA followed by Scheffe’s post-hoc test. Data are presented as mean ± SEM. (C) CAMDI KO mice inhibited cLTP-induced cell surface expression of AMPAR. Antibody-feeding assay was performed on hippocampal neurons from WT and CAMDI KO mice. Neurons were stimulated with or without glycine and incubated. Scale bar, 5 μm. (D) Cell surface expression of GluA2 subunit on individual neurons from (C). The average GluA2 fluorescence intensity was normalized to the dendritic length (10 μm) for each experimental condition. Control, Gly-, n = 36; Control, Gly+, n = 31; CAMDI KO, Gly-, n = 26; CAMDI KO, Gly+, n = 26. ***, p<0.001, Two-way ANOVA followed by Scheffe’s post-hoc test. Data are presented as mean ± SEM.

AMPA receptors are transported from recycling endosomes to the plasma membrane for LTP [4]. Therefore, we next examined the role of CAMDI in the activity-dependent cell surface expression of AMPARs into synapses during LTP. After glycine-induced chemical LTP (c-LTP), cell surface expression of GluA2 subunit was significantly greater than in unstimulated control cells (Fig 4A and 4B). In contrast, the increase in GluA2 receptor was strongly blocked in CAMDI-sh-transfected hippocampal neurons (Fig 4A and 4B). This inhibition of the increase of GluA2 subunit was completely rescued by co-transfection with CAMDI-sh and CAMDI-resi, a construct resistant to this shRNA (Fig 4A and 4B). These results indicated that CAMDI regulated LTP-induced cell surface expression of GluA2 subunit in hippocampal neurons.

To confirm this observation, c-LTP-induced GluA2 surface expression assay was performed using primary cultured hippocampal neurons derived from WT mice and CAMDI KO mice. c-LTP-induced GluA2 cell surface expression increased in WT neurons, whereas a significant decrease in surface GluA2 subunit expression was observed in CAMDI-deficient neurons (Fig 4C and 4D). Taken together, these results indicated that CAMDI regulated LTP-induced cell surface expression of GluA2 subunit in hippocampal neurons.

### CAMDI interacts with KIBRA

We performed a yeast two-hybrid screening of a mouse brain cDNA library by using the CAMDI coiled-coil region as a bait. We identified a KIBRA fragment containing a C-terminal coiled-coil region (863 to 1105 amino acids). KIBRA forms a complex with GluA2 subunit by interacting with PICK1, and KIBRA knockdown accelerates the rate of GluA2 subunit recycling by decreasing the retention of GluA2 receptor in the cytoplasmic pool [11], suggesting the involvement of CAMDI–KIBRA interaction in the regulation of GluA2 subunit recycling.

**Fig 5:**
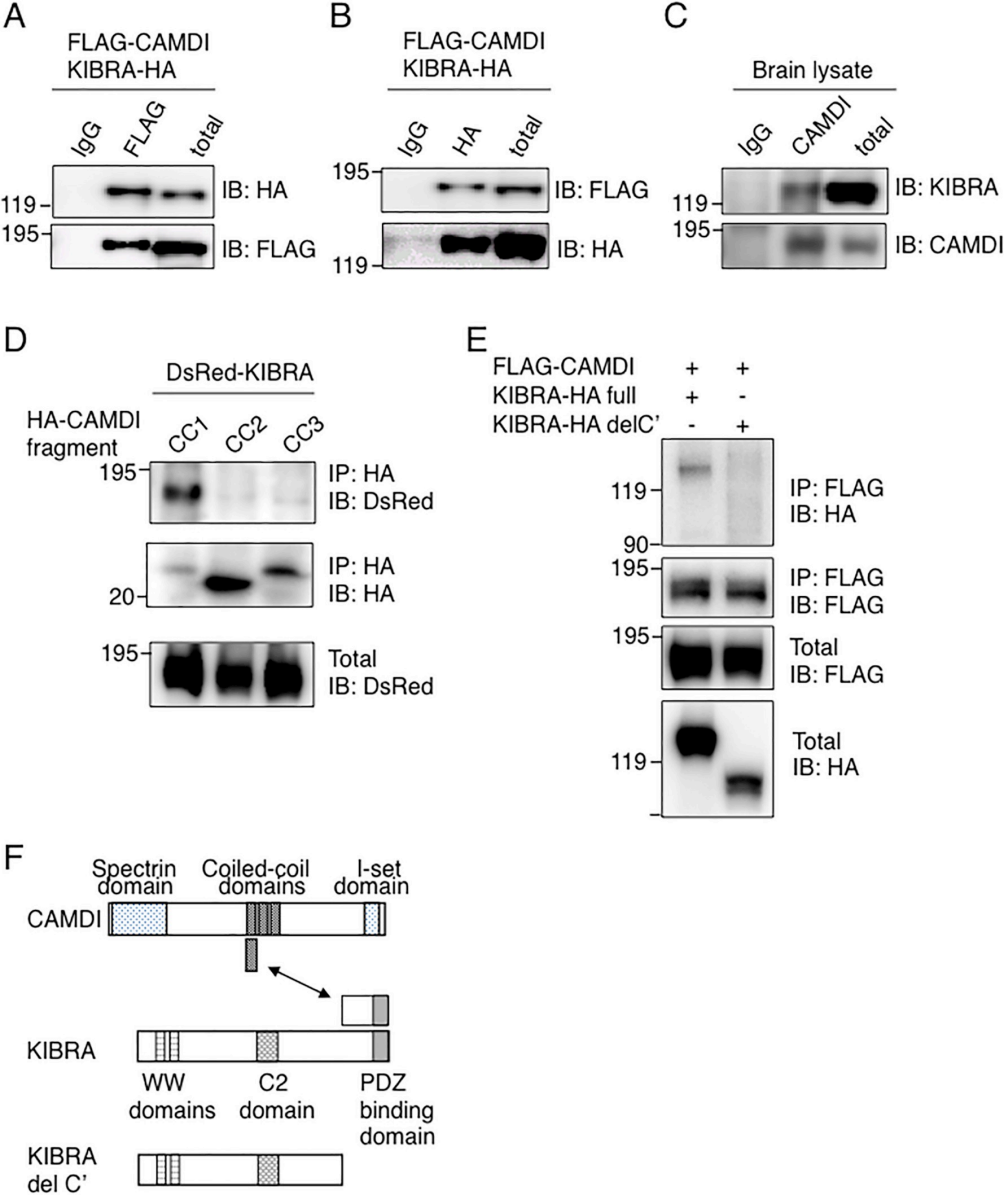
Interaction of CAMDI with KIBRA. (A, B) Interaction of FLAG-CAMDI with KIBRA-HA. Immunoprecipitation followed by immunoblot (IP-IB) assays were performed with the indicated antibodies. n = 3 independent experiments. (C) Endogenous interaction of CAMDI with KIBRA in the mouse brain. IP-IB assays were performed with the indicated antibodies using P56 brain lysate. n = 3 independent experiments. (D) First coiled-coil domain of CAMDI responsible for interaction with KIBRA. Three HA-tagged CAMDI coiled-coil domains (CC1, 576–706; CC2, 707–906; CC3, 907–1,106 amino acids) and DsRed-KIBRA were co-transfected and subjected to IP-IB assay using indicated antibody. n = 3 independent experiments. (E) CAMDI interacts with C-terminal region of KIBRA. FLAG-CAMDI and KIBRA-HA full or KIBRA-HA deletion mutant (990–1,104 amino acids deletion) were co-transfected and subjected to IP-IB assay using indicated antibody. n = 3 independent experiments. (F) Schematic illustration of interaction between CAMDI, KIBRA and KIBRA del C’.

To confirm the interaction of CAMDI with KIBRA, immunoprecipitation and western blot (IP-IB) analysis was performed on SH-SY5Y cells co-expressing FLAG-CAMDI and KIBRA-HA. Expectedly, KIBRA-HA and FLAG-CAMDI were co-immunoprecipitated (Fig 5A and 5B). Interaction of endogenous CAMDI and KIBRA was detected with anti-KIBRA antibody in WT mouse brain, but not in CAMDI KO mouse brain (Fig 5C and S3A, S3C, S3D, S3E, S3F and S4 Figs). To determine the interaction domain of CAMDI with KIBRA, we constructed three fragments of the CAMDI coiled-coil region. IP-IB analysis indicated that the first coiled-coil domain in CAMDI interacted with KIBRA (Fig 5D). On the other hand, CAMDI failed to interact with a KIBRA mutant lacking the C-terminal region of KIBRA (Fig 5E); this was consistent with the results of the yeast two-hybrid screening. Thus, the first coiled-coil domain in CAMDI specifically interacted with the C-terminal region of KIBRA (Fig 5F). Immunofluorescence analysis revealed the partial co-localization of CAMDI and KIBRA as vesicle-like structures (S3B Fig), suggesting that CAMDI and KIBRA have roles in vesicle transport.

### CAMDI regulates Rab11 activation and interaction between KIBRA and GluA2

**Fig 6:**
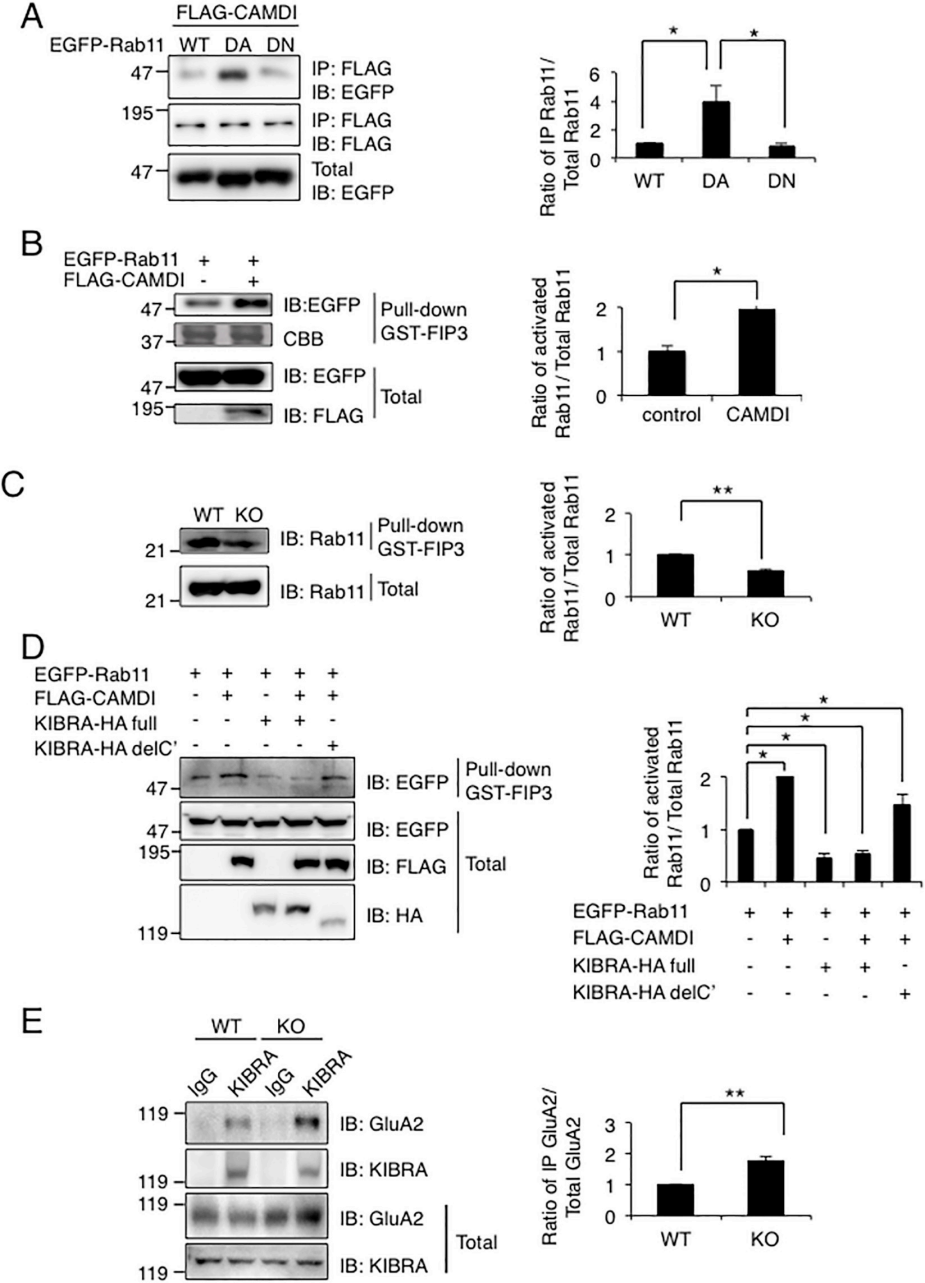
CAMDI regulates Rab11 activation and interaction between KIBRA and GluA2. (A) CAMDI preferentially interacts with active form of Rab11. FLAG-CAMDI and EGFP-Rab11a (WT, DA (dominant active), DN (dominant negative) were co-transfected and subjected to IP-IB assay using indicated antibody. n = 3 independent experiments. *, p<0.05, Student’s t-test. Data are presented as mean ± SEM. (B) Activation of Rab11 by CAMDI over-expression. FLAG-CAMDI and EGFP–Rab11a were co-transfected and subjected to pull-down assay using GST-FIP3. n = 3 independent experiments. *, p<0.05, Student’s t-test. Data are presented as mean ± SEM. (C) Attenuated activity of Rab11 in the CAMDI KO hippocampus. Pull-down assay using GST-FIP3 was performed on lysates from WT or CAMDI KO hippocampus. n = 3 independent experiments. **, p<0.01, Student’s t-test. Data are presented as mean ± SEM. (D) Inhibition of CAMDI-dependent Rab11 activation by KIBRA over-expression. Indicated plasmids were co-transfected and subjected to pull-down assay using GST-FIP3. n = 3 independent experiments. *, p<0.05, Student’s t-test. Data are presented as mean ± SEM. (E) Enhanced interaction between KIBRA and GluA2 receptor in CAMDI KO mice. IP-IB assays were performed with the indicated antibodies in P56 brain lysate. n = 3 independent experiments. **, p<0.01, Student’s t-test. Data are presented as mean ± SEM.

Rab11 is a recycling endosome marker and is associated with the endocytic recycling system [21]. We confirmed the partial co-localization of FLAG-CAMDI with EGFP-Rab11 and KIBRA-HA (S5A Fig). We found that CAMDI preferentially interacted with the dominant active form of Rab11 (Fig 6A and S5B Fig). Furthermore, GST-pull-down assay using the Rab11-interacting domain of Rab11–FIP3 demonstrated that CAMDI overexpression activated Rab11 (Fig 6B and S5C Fig). Consistently, Rab11 was markedly downregulated in the CAMDI-deficient hippocampus (Fig 6C). The decrease in active Rab11 seen in the CAMD1 KO mice is consistent with the increase in internalized AMPA receptors seen in the CAMDI KO mice since active Rab11 is important in recycling of AMPA receptors [4].

KIBRA is involved in the trafficking of AMPAR [11]. KIBRA knockdown does not affect the internalization of AMPAR, but it accelerates the rate of GluA2 recycling to the membrane. We therefore hypothesized that KIBRA would antagonize the function of CAMDI in AMPAR recycling, and we examined the effect of KIBRA on CAMDI-mediated Rab11 activation. As expected, KIBRA overexpression inhibited CAMDI-mediated Rab11 activation (Fig 6D). KIBRA forms a complex with GluA2 subunit *in vivo* [11]. We observed an increase in KIBRA–GluA2 interaction in the CAMDI-deficient hippocampus (Fig 6E), suggesting that enhanced KIBRA–GluA2 interaction as a result of CAMDI deletion retains AMPAR in the cytoplasmic compartment after internalization. Taken together, these results suggest that CAMDI and KIBRA have opposing roles in Rab11-mediated AMPAR cell surface expression.

## Discussion

Cognitive psychology has become an important discipline in the study of a number of psychiatric disorders, including developmental disorders [18]. To date, however, no report has directly explained the common molecular mechanisms underlying psychiatric disorders and memory disturbance. CAMDI is a factor associated with psychiatric behaviors in mice. To search for the precise role of CAMDI in the brain, we performed a yeast two-hybrid screening and identified KIBRA as a novel CAMDI-interacting protein, suggesting that CAMDI plays a role in AMPAR recycling and consequently in learning and memory. Several lines of evidence indicate that there are alterations in AMPAR trafficking in psychiatric disorders [22, 23]. Here, we suggest that CAMDI KO mice had impaired learning and memory performance, perhaps partly via dysregulation of AMPAR recycling. Although there are more remaining intracellular AMPARs at 60 min (Fig 3C and 3D), there are less surface AMPARs under basal conditions (S2 Fig). These data suggest that CAMDI regulates the rate of AMPAR recycling. To our knowledge, this provides the first evidence that CAMDI links psychiatric behaviors and cognitive deficits in mice. However, since CAMDI KO mice had the deficits during neuronal development, memory deficits can be attributed to errors of development. In addition, we could not deny the possibility of an increased degradation of AMPA receptor. As KIBRA KO mice showed developmental abnormalities in both LTP and LTD [11, 24], KIBRA may function in a context-dependent manner and the mechanisms by which KIBRA regulates LTP and AMPAR trafficking remains unknown. Further analysis of CAMDI KO should improve our understanding of the common molecular mechanisms underlying the relationship between psychiatric disorders and memory disturbance.

CAMDI-interacting protein, MRLC 2a (myosin regulatory light chain 2a), seems to be of great importance in this pathway. CAMDI preferentially interacts with the phosphorylated form of MRLC 2a and stabilizes it. Indeed, the actin cytoskeleton influences AMPAR trafficking by regulating the local actin environment in the dendritic spine [25, 26]. Therefore, CAMDI may regulate AMPAR recycling through remodeling of the actin cytoskeleton via myosin II. Given that CAMDI controls microtubule remodeling through centrosomal stabilization by HDAC6 inhibition [15], CAMDI may act at the intersection of the forces of the microtubules and the actin cytoskeleton. It could be possible that dysfunction of protein(s) acting on both the actin and microtubule cytoskeletons may link impaired memory performance and developmental or psychiatric disorders [14].

KIBRA is associated with human memory performance [27] and plays a pivotal role in the regulation of AMPAR recycling [11]. Here, we identified a novel association of KIBRA as a CAMDI-interacting protein. Although KIBRA retains recycling endosomes in the cytosolic compartment, the molecular mechanism by which this occurs is not fully elucidated. We demonstrated here that CAMDI promotes LTP-dependent AMPAR cell surface expression. In addition, CAMDI binds KIBRA and regulates interaction of KIBRA and AMPAR. Furthermore, KIBRA inhibited CAMDI-induced Rab11 activation, but a KIBRA mutant lacking the C-terminal region, which is the CAMDI-binding domain, failed to inhibit CAMDI-induced Rab11 activation. Thus, KIBRA may retain recycling endosomes in the cytosolic compartment, at least in part, through the inhibition of CAMDI by direct interaction. On the other hand, we observed an enhanced interaction of KIBRA with GluA2 subunit in the CAMDI-deficient hippocampus (see Fig 6E). KIBRA interacts with PICK1, resulting in retention of the GluA2 subunit in intracellular pools and inhibiting their recycling to the plasma membrane [28, 29]. Therefore, it is also possible that CAMDI inhibits KIBRA interaction with PICK1, thereby releasing the KIBRA–PICK1-mediated restraint of the GluA2 subunit in the intracellular pools. CAMDI and KIBRA therefore control each other’s function. Further experiments using KIBRA C-terminal deletion mutant, which includes PICK1 binding site, are needed to examine the possibility of involvement of PICK1 in KIBRA function.

Accumulating evidence suggests that protein kinase M zeta (PKMζ) is involved in the regulation of KIBRA function in learning and memory. PKMζ increases the number of active AMPARs at the post-synapse by promoting AMPAR trafficking during memory consolidation [30, 31]. KIBRA is a substrate for PKMζ [32], suggesting that PKMζ antagonizes KIBRA function by direct phosphorylation. Thus, we hypothesize that phosphorylated KIBRA by PKMζ releases CAMDI, allowing CAMDI-dependent Rab11 activation and consequent promotion of AMPR recycling. Further studies are needed to clarify the detailed molecular mechanism underlying CAMDI regulation by KIBRA. In conclusion, KIBRA phosphorylation may regulate CAMDI function.

Further analysis of the molecular mechanisms underlying CAMDI-mediated AMPAR trafficking will contribute greatly to the elucidation of learning and memory disturbance in developmental and psychiatric disorders.

## Supporting information

S1 Fig (A) In training session, there was no significant difference in interest to objects between WT and CAMDI KO mice. N. S., not significant. Two-way ANOVA followed by Scheffe’s post-hoc test. Data are presented as mean ± SEM. (B) Ratio of trial 3 / trial 1. n = 9 for WT mice, n = 9 for KO mice. *, p<0.05, Student’s t-test. Data are presented as mean ± SEM.
(TIFF)

S2 Fig No difference in internalized receptor at 0min between WT mice and CAMDI KO hippocampal neurons. Control, n = 29; CAMDI KO, n = 32. N. S., not significant. Student’s t-test. Data are presented as mean ± SEM.
(TIFF)

S3 Fig (A) Specificity of anti-KIBRA antibody. Cells were transfected with KIBRA-HA plasmid and cell lysate was provided to immunoblot assay. (B) Co-localization of FLAG-CAMDI and KIBRA-HA. SH-SY5Y cells were transfected with indicated plasmids and subjected to immunocytochemistry with antibodies against FLAG (green) and HA (red). The correlation between CAMDI and KIBRA was (0.69 ± 0.06, 10 cells). Scale bar, 5 μm. (C, D) Inhibitory effect of KIBRA-sh3 on KIBRA expression in HEK293 cells (C) and primary hippocampal neurons (D). (E) Quantification of the KIBRA knockdown effect from (D). n = 3 independent experiments. ***, p<0.001, Student’s t-test. Data are presented as mean ± SEM. (F) Specificity of anti-KIBRA antibody was validated by KIBRA knock down. Hippocampal neurons were transfected with KIBRA-sh3 and EGFP plasmids at DIV1 and subjected to immunocytochemistry with antibodies against EGFP (green) and KIBRA (red) at DIV3. Line scan analyses revealed anti-KIBRA antibody works in immunocytochemistry.
(TIFF)

S4 Fig IP-IB assay with CAMDI antibody in CAMDI KO lysate to confirm specificity. (TIFF)

S5 Fig (A) FLAG-CAMDI co-localized with EGFP-Rab11. SH-SY5Y cells were co-transfected with indicated plasmids and subjected to immunocytochemistry with antibodies against EGFP (green) and FLAG (red). The correlation between CAMDI and Rab11 was (0.75 ± 0.07, 10 cells). Scale bar, 5 μm. (B) CAMDI interacts with Rab11. FLAG-CAMDI and EGFP-Rab11 were co-transfected and subjected to IP-IB assay using indicated antibody. n = 3 independent experiments. (C) Activated Rab11 binds GST-FIP3 (C’ 20 a.a. of Rab11-FIP3). GST or GST–FIP3 was immobilized on glutathione-Sepharose and then tested for its ability to bind EGFP-Rab11 in SH-SY5Y cell lysate and subjected to IB assay using indicated antibody. n = 3 independent experiments.
(TIFF)
