# Supplementary figures and images for: CAMDI interacts with the human memory-associated protein KIBRA and regulates AMPAR cell surface expression and cognition
Source: PLoS One. 2019 Nov 15;14(11):e0224967. doi: 10.1371/journal.pone.0224967 (PMC6857912; doi:10.1371/journal.pone.0224967)

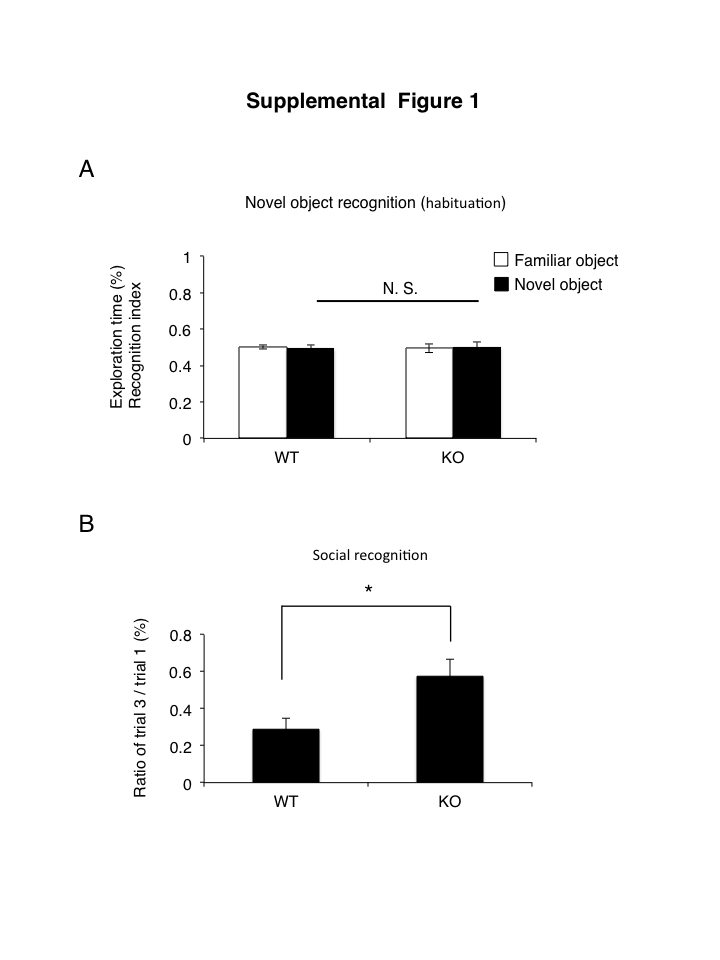

Supplement: S1 Fig — (A) In training session, there was no significant difference in interest to objects between WT and CAMDI KO mice. N. S., not significant. Two-way ANOVA followed by Scheffe’s post-hoc test. Data are presented as mean ± SEM. (B) Ratio of trial 3 / trial 1. n = 9 for WT mice, n = 9 for KO mice. *, p<0.05, Student’s t-test. Data are presented as mean ± SEM. (TIFF) [file pone.0224967.s001.tiff]

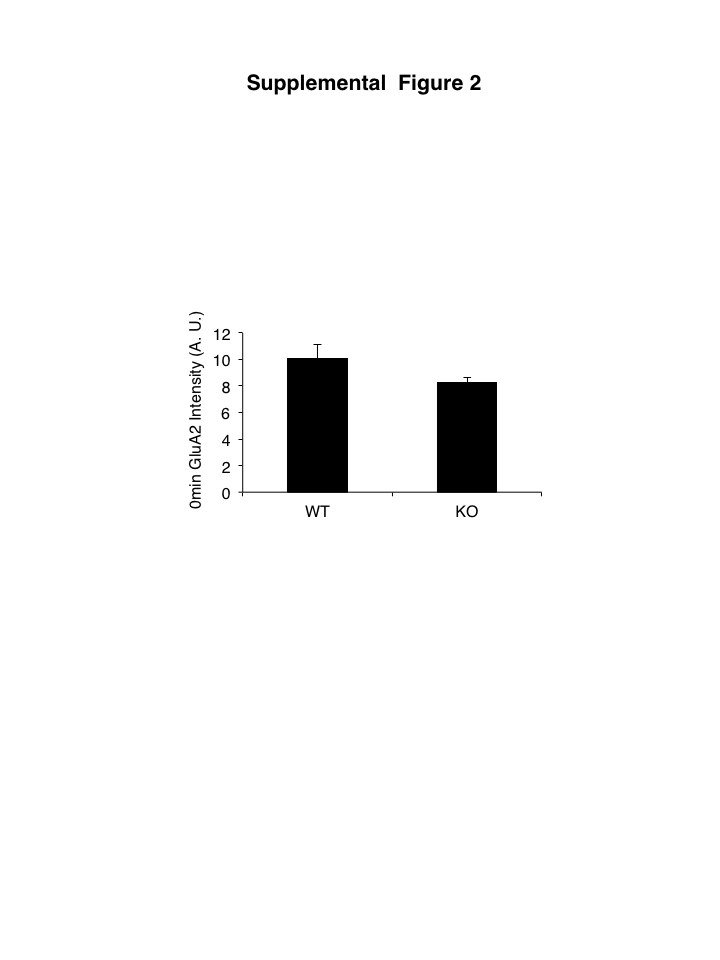

Supplement: S2 Fig — Control, n = 29; CAMDI KO, n = 32. N. S., not significant. Student’s t-test. Data are presented as mean ± SEM. (TIFF) [file pone.0224967.s002.tiff]

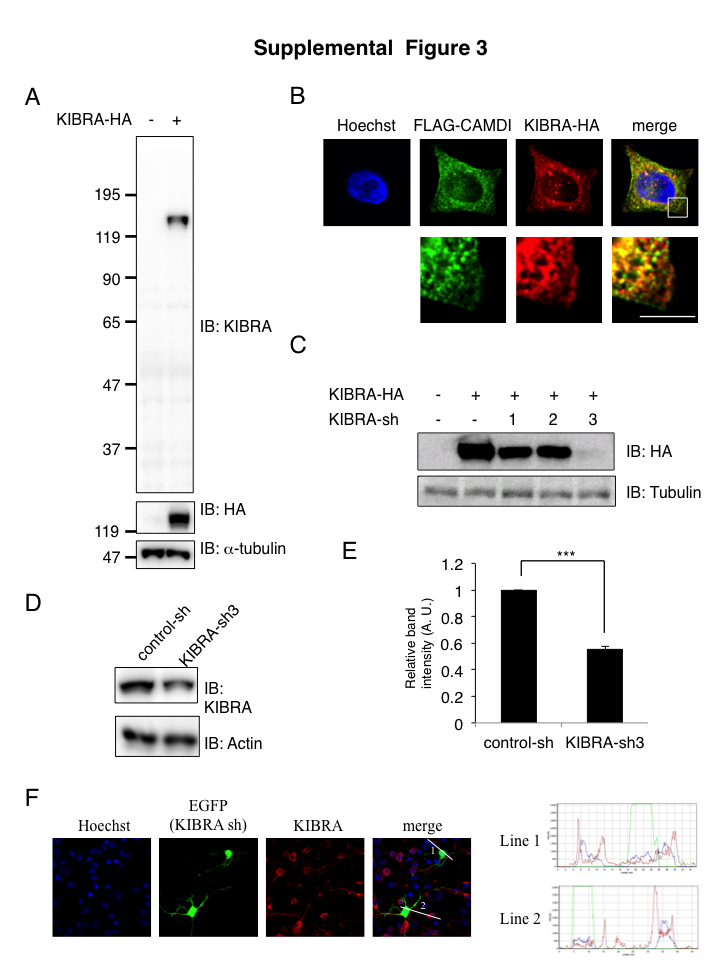

Supplement: S3 Fig — (A) Specificity of anti-KIBRA antibody. Cells were transfected with KIBRA-HA plasmid and cell lysate was provided to immunoblot assay. (B) Co-localization of FLAG-CAMDI and KIBRA-HA. SH-SY5Y cells were transfected with indicated plasmids and subjected to immunocytochemistry with antibodies against FLAG (green) and HA (red). The correlation between CAMDI and KIBRA was (0.69 ± 0.06, 10 cells). Scale bar, 5 μm. (C, D) Inhibitory effect of KIBRA-sh3 on KIBRA expression in HEK293 cells (C) and primary hippocampal neurons (D). (E) Quantification of the KIBRA knockdown effect from (D). n = 3 independent experiments. ***, p<0.001, Student’s t-test. Data are presented as mean ± SEM. (F) Specificity of anti-KIBRA antibody was validated by KIBRA knock down. Hippocampal neurons were transfected with KIBRA-sh3 and EGFP plasmids at DIV1 and subjected to immunocytochemistry with antibodies against EGFP (green) and KIBRA (red) at DIV3. Line scan analyses revealed anti-KIBRA antibody works in immunocytochemistry. (TIFF) [file pone.0224967.s003.tiff]

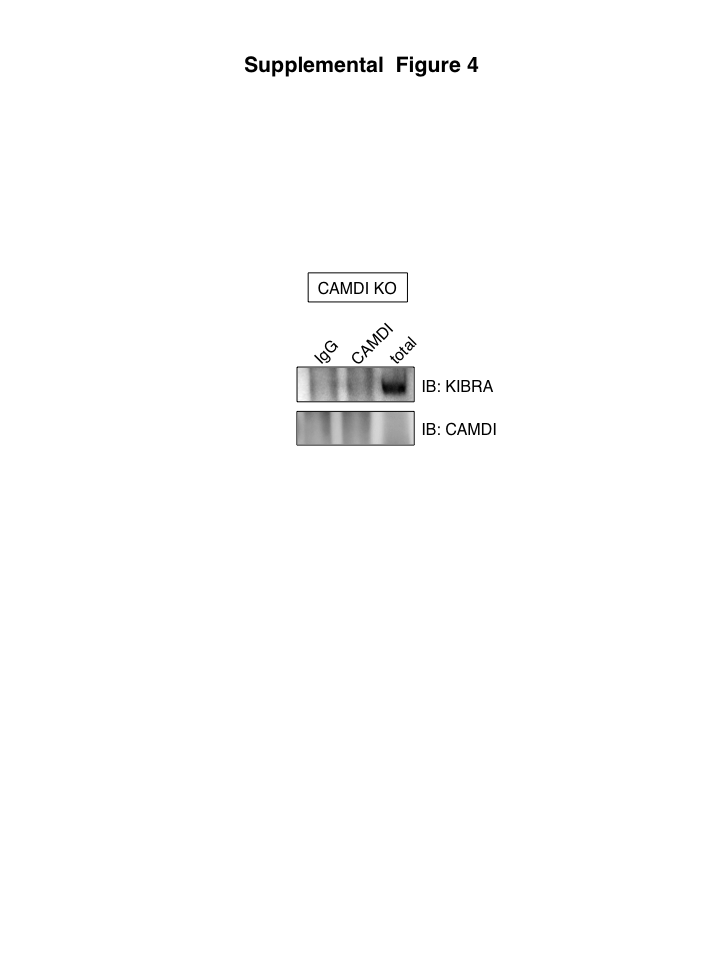

Supplement: S4 Fig — (TIFF) [file pone.0224967.s004.tiff]

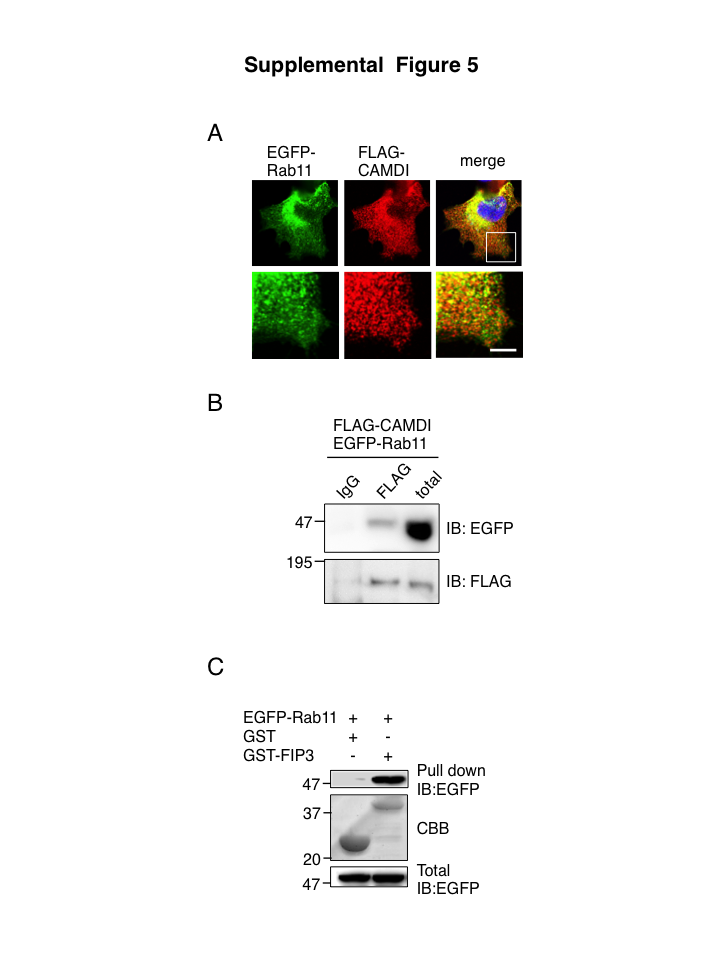

Supplement: S5 Fig — (A) FLAG-CAMDI co-localized with EGFP-Rab11. SH-SY5Y cells were co-transfected with indicated plasmids and subjected to immunocytochemistry with antibodies against EGFP (green) and FLAG (red). The correlation between CAMDI and Rab11 was (0.75 ± 0.07, 10 cells). Scale bar, 5 μm. (B) CAMDI interacts with Rab11. FLAG-CAMDI and EGFP-Rab11 were co-transfected and subjected to IP-IB assay using indicated antibody. n = 3 independent experiments. (C) Activated Rab11 binds GST-FIP3 (C’ 20 a.a. of Rab11-FIP3). GST or GST–FIP3 was immobilized on glutathione-Sepharose and then tested for its ability to bind EGFP-Rab11 in SH-SY5Y cell lysate and subjected to IB assay using indicated antibody. n = 3 independent experiments. (TIFF) [file pone.0224967.s005.tiff]
